# Plasma Atherogenic Index Predicts Malignant Arrhythmias in Non–ST-Elevation Myocardial Infarction

**DOI:** 10.5152/eurasianjmed.2026.251315

**Published:** 2026-04-10

**Authors:** Alperen Taş, Muhammet Salih Ateş, Çağatay Tunca

**Affiliations:** Department of Cardiology, Ahi Evran University Training and Research Hospital, Kırşehir, Türkiye

**Keywords:** Arrhythmia, non ST-elevation myocardial infarction, percutaneous coronary intervention

## Abstract

**Background:**

Malignant ventricular arrhythmias are serious complications of non–ST-elevation myocardial infarction. The plasma atherogenic index, derived from triglyceride and high-density lipoprotein cholesterol, reflects atherogenic dyslipidemia. Its role in predicting malignant arrhythmias is unclear. The primary aim of this study was to determine whether the plasma atherogenic index independently predicts malignant ventricular arrhythmias in patients with non–ST-elevation myocardial infarction.

**Methods:**

A total of 502 patients with non–ST-elevation myocardial infarction admitted between January 2024 and January 2025 were retrospectively analyzed. Patients were grouped by arrhythmia occurrence and tertiles of the plasma atherogenic index. Logistic regression identified predictors of arrhythmias. Cox regression identified predictors of malignant arrhythmias. Receiver operating characteristic analysis assessed discriminative performance, and Kaplan–Meier analysis evaluated survival.

**Results:**

Malignant ventricular arrhythmias occurred in 47 patients (9.4%). They had significantly higher plasma atherogenic index values (0.48 ± 0.35 vs. 0.21 ± 0.29, *P* < .001). In multivariable analysis, multivessel disease, neutrophil, troponin T, low-density lipoprotein cholesterol, reduced ejection fraction, and the plasma atherogenic index independently predicted arrhythmias. For mortality, no-reflow, troponin T, malignant arrhythmia, and the plasma atherogenic index (hazard ratio 1.342, 95% confidence interval 1.211-1.487, *P* = .006) were independent predictors. The plasma atherogenic index had the highest discriminative value (area under the curve 0.721 for arrhythmias; 0.709 for mortality) with a cut-off of 0.32 (sensitivity 68%, specificity 68%). Kaplan–Meier analysis showed lower survival in the highest tertile.

**Conclusion:**

Elevated plasma atherogenic index independently predicts malignant arrhythmias and short-term mortality in non–ST-elevation myocardial infarction. As a simple, widely available lipid marker, it may improve risk stratification in high-risk patients.

**Main Points**

- Plasma atherogenic index (PAI) is an independent predictor of malignant ventricular arrhythmias in patients with non–ST-elevation myocardial infarction (NSTEMI), even after adjustment for clinical severity, myocardial injury markers, and left ventricular function.
- Higher PAI values are strongly associated with increased in-hospital mortality, demonstrating prognostic value comparable to or greater than traditional biomarkers.
- Patients in the highest PAI tertile had markedly higher rates of malignant arrhythmias and in-hospital mortality, indicating that PAI effectively stratifies early risk in NSTEMI.
- Plasma atherogenic index is an inexpensive, easily obtainable lipid-based marker derived from routine laboratory testing, making it a practical tool for early risk stratification and monitoring in acute coronary syndromes

## Introduction

Malignant arrhythmias, including sustained ventricular tachycardia and ventricular fibrillation, are among the most severe and life-threatening complications observed in patients with acute coronary syndromes (ACS) and myocardial infarction.^1^ These arrhythmias are associated with a markedly increased risk of sudden cardiac death, prolonged hospitalization, and poor clinical outcomes.^2^ Identifying high-risk patients during the early phases of hospitalization is critical for timely implementation of intensive monitoring and preventive strategies, thereby reducing mortality rates.^3^

The pathogenesis of malignant arrhythmias is multifactorial, involving ischemia-induced myocardial electrical instability, autonomic nervous system imbalance, myocardial remodeling, and systemic inflammatory responses.^4^^,^^5^ Increasing evidence also suggests that metabolic disturbances, particularly those related to lipid metabolism, play a crucial role in arrhythmogenesis by promoting atherosclerosis, plaque rupture, and microvascular dysfunction.^6^^,^^7^ Dyslipidemia, characterized by elevated triglyceride (TG) and low high-density lipoprotein cholesterol (HDL-C) levels, has been identified as a strong predictor of adverse cardiovascular events, including arrhythmic complications.^8^^,^^9^

In recent years, the plasma atherogenic index (PAI), calculated as the base-10 logarithm of the TG/HDL-C molar ratio, has gained attention as a novel, integrative marker of atherogenic dyslipidemia.^10^ It has been demonstrated to correlate with metabolic syndrome, insulin resistance, and the severity of coronary artery disease, making it a practical and cost-effective biomarker for cardiovascular risk stratification.^11^

Multiple studies have linked elevated PAI levels with poor outcomes in ACS, including increased infarct size, impaired left ventricular function, and higher rates of in-hospital complications.^12^ Although PAI is an established marker of adverse cardiovascular prognosis, evidence linking it to malignant arrhythmias during the acute phase of myocardial infarction remains limited. Because PAI integrates both pro- and anti-atherogenic lipid fractions, it may better reflect lipid-related arrhythmogenic risk than individual lipid parameters. Based on this rationale, it was hypothesized that higher PAI levels would be independently associated with malignant ventricular arrhythmias. Focus was specifically placed on NSTEMI, a heterogeneous but pathophysiologically consistent subgroup in which ischemic burden, multivessel disease, and arrhythmic susceptibility vary widely; including the entire ACS spectrum could introduce excess heterogeneity and obscure true associations. Therefore, the primary objective of this study was to evaluate whether plasma atherogenic index independently predicts malignant ventricular arrhythmias in NSTEMI patients.

## Material and Methods

### Study Population

**Figure 1.**
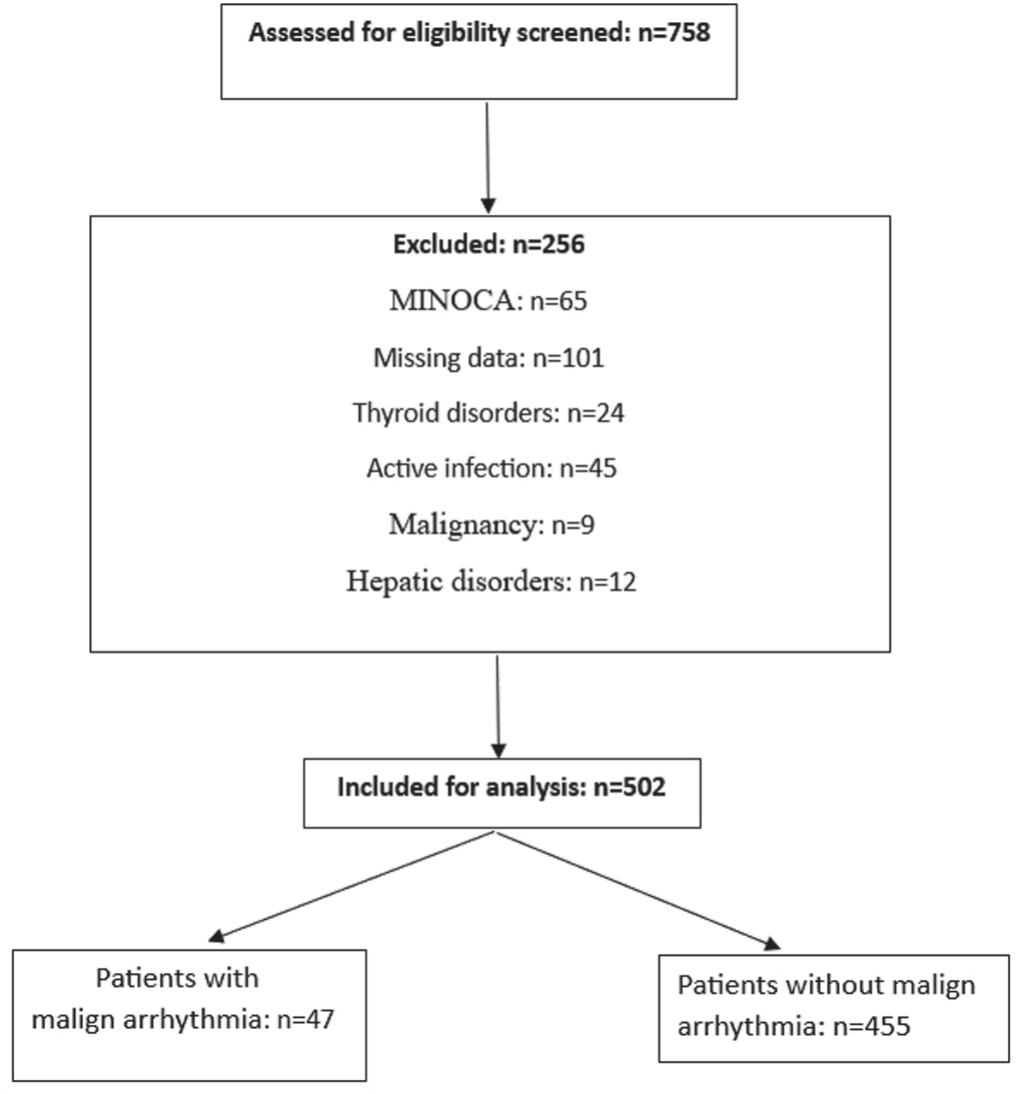
Study flow diagram illustrating patient selection, exclusions, and final analytic cohort.

This retrospective observational study included consecutive NSTEMI patients admitted to the coronary care unit of the tertiary cardiovascular center between January 2024 and January 2025 for coronary angiography. Non–ST-elevation myocardial infarction was defined according to ESC guidelines based on ischemic symptoms, ECG findings without persistent ST elevation, and elevated or dynamic high-sensitivity troponin levels.^13^ Patients with MINOCA (<50% stenosis in all major epicardial vessels), missing data, active infection, malignancy, or known thyroid or hepatic disorders were excluded. The study flowchart is presented in Figure 1. The study was approved by the Ahi Evran University ethics committee and conducted in accordance with the Declaration of Helsinki. Written informed consent was obtained from the patients who agreed to take part in the study.

### Blood Sample Collection and Biochemical Analysis

Venous blood samples were obtained within the first 24 hours of admission after an overnight fast of at least 8 hours, before any coronary intervention. Blood was collected in standard EDTA and plain gel tubes, centrifuged at 3000 rpm for 10 minutes, and analyzed immediately or stored at –80°C. Serum TG, HDL-C, and LDL-C levels were measured using standard enzymatic colorimetric methods on an automated analyzer. The plasma atherogenic index was calculated as the base-10 logarithm of the molar TG/HDL-C ratio.^14^

### Definition of Comorbidities

Diabetes mellitus was defined according to ADA criteria as a prior diagnosis, use of oral antidiabetic agents or insulin, or admission measurements showing fasting plasma glucose ≥126 mg/dL or HbA1c ≥6.5%.^15^ Hypertension was identified based on a known history of hypertension, current antihypertensive therapy, or repeated in-hospital blood pressure readings ≥140/90 mmHg in accordance with ESC guidelines.^16^ Smoking status was classified as current smoking within the past 12 months, former smoking if cessation exceeded 12 months, and never smoking for individuals with no prior smoking history.

### Coronary Angiography and Percutaneous Coronary Intervention

All NSTEMI patients underwent diagnostic coronary angiography via radial or femoral access using the standard Judkins technique. Decisions regarding percutaneous coronary intervention (PCI) or coronary artery bypass grafting (CABG) were made by the interventional cardiologist based on clinical and angiographic findings. For patients undergoing PCI, balloon angioplasty and/or stent implantation were performed according to contemporary practice, and periprocedural antiplatelet and anticoagulant therapies were administered in line with current ESC ACS guidelines. The presence of no-reflow was defined angiographically as TIMI flow grade ≤2 after PCI without mechanical obstruction, dissection, or spasm.^17^

### Outcome Definition

The study evaluated 2 clinically relevant outcomes: malignant ventricular arrhythmias and in-hospital mortality. Malignant ventricular arrhythmias were defined as sustained ventricular tachycardia (VT) or ventricular fibrillation (VF) occurring during the peri-procedural period. Sustained VT was defined as VT lasting ≥30 seconds or requiring urgent termination due to hemodynamic instability. In-hospital mortality was defined as death from any cause during the index hospitalization. Continuous ECG monitoring in the CCU and catheterization laboratory ensured accurate detection and documentation of arrhythmic events.

### Statistical Analysis

Statistical analyses were performed using IBM SPSS Statistics for Windows, Version 22.0 (IBM SPSS Corp.; Armonk, NY, USA). Continuous variables were assessed for normality (Kolmogorov–Smirnov) and reported as mean ± SD or median (min–max), while categorical variables were presented as counts and percentages. Group comparisons (malignant arrhythmia vs. no arrhythmia) used the t-test or Mann–Whitney *U-*test for continuous variables and the chi-square or Fisher’s exact test for categorical data.

Patients were additionally stratified by PAI tertiles, with comparisons across groups performed using 1-way ANOVA or Kruskal–Wallis tests and *chi*-square tests for categorical variables; Bonferroni-adjusted post-hoc tests identified pairwise differences.

Univariable logistic regression identified factors associated with malignant arrhythmia, and variables with *P* < .05 or clinical relevance were entered into multivariable models; results are expressed as OR with 95% CI. In-hospital mortality was evaluated with Cox proportional hazards regression, with significant univariable parameters included in the multivariable model; hazard ratios (HR) with 95% CI were reported. Multicollinearity was assessed using variance inflation factors.

Receiver-operating characteristic (ROC) curves and area under the curve (AUC) values were used to assess predictive performance, with optimal PAI cut-off identified by the Youden index. Survival was evaluated using Kaplan–Meier analysis and compared with the log-rank test.

## Results

A total of 502 patients were analyzed; 47 (9.4%) developed malignant ventricular arrhythmias and 455 (90.6%) did not. Among the 47 arrhythmic events, 40 (85.1%) were sustained VT and 7 (14.9%) VF; 5 episodes (10.6%) terminated with IV amiodarone, whereas 42 (89.4%) required direct current cardioversion. In-hospital mortality occurred in 41 patients (8.2%). Baseline clinical, laboratory, and procedural characteristics of patients with and without malignant arrhythmias are shown in Table 1. Age, sex, hypertension, prior PCI, statin use, and most laboratory parameters did not differ significantly between groups (all *P* > .05).

However, malignant arrhythmia was significantly more frequent in patients with diabetes mellitus (44.7% vs. 25.7%, *P* = .006) and chronic renal failure (10.6% vs. 4.2%, *P* = .048). These patients also had lower systolic (123.85 ± 37.18 vs. 137.63 ± 30.27 mmHg, *P* = .007) and diastolic blood pressure (71.02 ± 19.55 vs. 77.72 ± 17.33 mmHg, *P* = .020) but higher heart rate (93.37 ± 30.89 vs. 82.11 ± 19.99 bpm, *P* = .001). Clinical severity was greater in the malignant arrhythmia group, as evidenced by a higher proportion of Killip class >1 (36.2% vs. 7.6%, *P* < .001), greater prevalence of multivessel coronary artery disease (48.9% vs. 31.4%, *P* = .015), and more frequent post-procedural no-reflow (21.3% vs. 7.3%, *P* = .001).

Metabolic and biochemical differences were marked. Patients with malignant arrhythmia had higher fasting glucose (176 vs. 128 mg/dL, *P* < .001), HbA1c (7.76 ± 1.96% vs. 6.74 ± 2.20%, *P* = .008), creatinine (1.04 vs. 0.84 mg/dL, *P* = .009), LDL-C (150 vs. 117 mg/dL, *P* = .001), triglycerides (234 vs. 132 mg/dL, *P* < .001), and neutrophil count (9.94 vs. 8.50 ×10^9^/L, *P* = .009), while HDL-C was significantly lower (26 vs. 36 mg/dL, *P* < .001). Importantly, the PAI was markedly higher in the malignant arrhythmia group (0.48 ± 0.35 vs. 0.21 ± 0.29, *P* < .001). Echocardiographically, left ventricular ejection fraction (LVEF) was reduced in these patients (35.42 ± 7.71% vs. 41.61 ± 8.75%, *P* < .001). In-hospital mortality was significantly greater among those with malignant arrhythmia (34% vs. 5.5%, *P* < .001).

When patients were stratified into PAI tertiles (27-75), several clinical and laboratory differences emerged (Table 2). The highest tertile had lower diastolic blood pressure, lower HDL-C, and higher triglyceride levels, along with a greater frequency of Killip class > I and post-procedural no-reflow. Malignant arrhythmias increased markedly across tertiles, peaking in the highest group (24.4% vs. 7.2% and 3.2%, *P* < .001). In-hospital mortality was likewise highest in this tertile (19.2% vs. 2.4% and 5.6%, *P* < .001).

Univariable analysis identified several predictors of malignant ventricular arrhythmia, including Killip class > 1, multivessel disease, post-procedural no-reflow, reduced eGFR, higher neutrophil count, elevated troponin T, LDL-C, increased PAI, and lower LVEF. In the multivariable model, the independent predictors were multivessel disease (OR: 2.726, 95% CI: 1.117-6.655, *P* = .028), neutrophil count (OR: 1.065, 95% CI: 1.015-1.118, *P* = .010), basal troponin T (OR: 1.000, 95% CI: 1.000-1.001, *P* = .010), LDL-C (OR: 1.023, 95% CI: 1.012-1.033, *P* < .001), PAI (OR: 1.228, 95% CI: 1.129-1.336, *P* < .001), and reduced LVEF (OR: 0.878, 95% CI: 0.824-0.934, *P* < .001) (Table 3).

In the univariable cox regression analysis for in-hospital mortality, lower systolic blood pressure (SBP), Killip class > I, post-procedural no-reflow, reduced eGFR, elevated troponin T, increased PAI, and the presence of malignant arrhythmia were significantly associated with mortality. In the multivariable model, post-procedural no-reflow (HR: 2.748, 95% CI: 1.075-7.025, *P* = .035), troponin T (HR: 1.000, 95% CI: 1.000-1.001, *P* = .009), PAI (HR: 1.342, 95% CI: 1.211-1.487, *P* = .006), and malignant arrhythmia (HR: 2.700, 95% CI: 1.014-7.193, *P* = .047) remained as independent predictors of mortality (Table 4).

**Figure 2.**
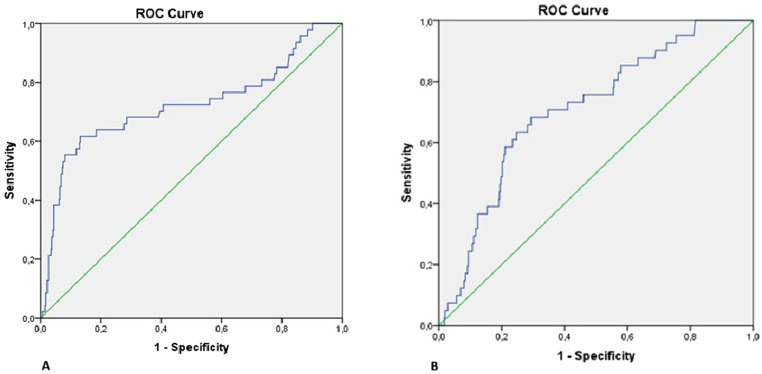
** A.** Receiver-operating characteristic (ROC) curve of PAI for predicting malignant arrhythmia **2 B.** Receiver-operating characteristic (ROC) curve of PAI for predicting mortality.

ROC analysis (Table 5, Figure 2A) demonstrated that PAI had the highest discriminative ability for predicting malignant arrhythmia (AUC: 0.721, 95% CI: 0.626-0.815, *P* < .001) with an optimal cut-off of 0.32, providing both sensitivity and specificity of 68%. The LVEF also showed good predictive capacity (AUC: 0.695, *P* < .001), followed by basal troponin T (AUC: 0.669, *P* < .001), LDL-C (AUC: 0.658, *P* = .001), and neutrophil count (AUC: 0.611, *P* = .021).

Receiver operating characteristic analysis further confirmed the prognostic utility of PAI for in-hospital mortality. The PAI showed the highest discriminative ability compared with troponin T (AUC: 0.709 vs. 0.644, both *P* < .05). The optimal cut-off value for PAI was 0.32, yielding a sensitivity of 68% and specificity of 68% (Table 5, Figure 2B).

**Figure 3.**
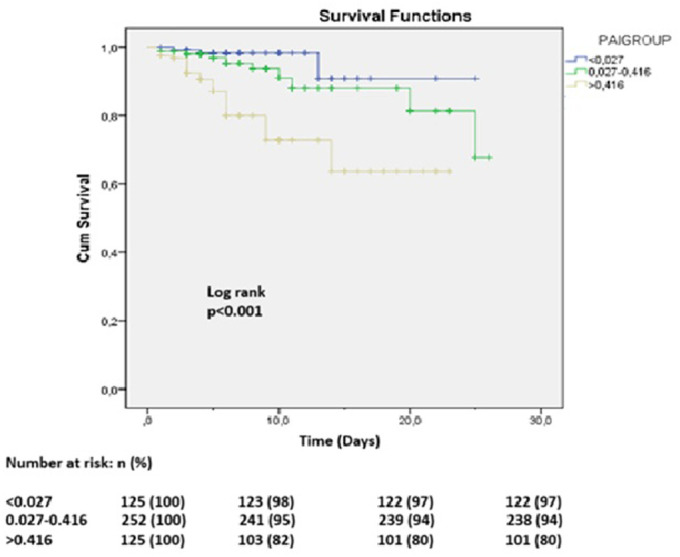
Kaplan–Meier survival curves according to tertiles of plasma atherogenic index (PAI: 27-75).

Kaplan–Meier survival analysis demonstrated that higher PAI levels were associated with worse overall survival (Figure 3). Patients in the highest tertile exhibited significantly reduced survival probability compared with those in the lower tertiles.

## Discussion

In this retrospective 1-year cohort of adults hospitalized with ACS, higher PAI was independently associated with malignant in-hospital ventricular arrhythmias after adjustment for clinical severity, myocardial injury, systolic function, and angiographic burden. These findings align with prior evidence supporting PAI as an integrated marker of atherogenic dyslipidemia and extend the existing literature by highlighting its relevance to electrical instability during the index admission rather than focusing solely on ischemic events or mortality.^18^ Beyond PAI, the multivariable pattern suggests a multidomain model of arrhythmic vulnerability: higher LDL-C reflects greater ischemic burden; elevated neutrophils and troponin indicate inflammation and acute necrosis; and multivessel disease with reduced LVEF denotes structural complexity and impaired reserve. The alignment of these domains with prior ACS literature linking hemodynamic stress, inflammation, and structural vulnerability to adverse electrophysiologic outcomes supports the plausibility of the findings.^19^ ^-^^22^ Notably, post-procedural no-reflow was related to arrhythmic events in univariable analysis but was not retained after full adjustment, consistent with partial mediation by infarct size, microvascular injury, and systolic dysfunction described in the no-reflow literature.^23^ ^-^^25^

The mechanistic plausibility of the PAI–arrhythmia link is strong. Elevated PAI reflects a constellation of triglyceride-rich lipoproteins and reduced HDL-mediated reverse cholesterol transport, and frequently coexists with a small dense LDL (sdLDL)–predominant phenotype.^26^ sdLDL particles penetrate the endothelium more easily, exhibit reduced affinity for LDL receptors, and are more prone to oxidative modification. These characteristics contribute to endothelial dysfunction, inflammation, and microvascular injury, which may in turn promote destabilization of ventricular electrophysiology during ischemia–reperfusion.^27^ This framework aligns with contemporary sdLDL reviews and with clinical data connecting atherogenic dyslipidemia to worse post-ACS trajectories, thereby offering a biologically coherent bridge between a lipid phenotype indexed by PAI and malignant arrhythmias.

Microvascular injury offers a plausible integrative pathway from an adverse lipid phenotype to electrical instability. Even after successful epicardial reperfusion, microvascular obstruction/no-reflow remains common and is associated with malignant arrhythmias and other poor outcomes.^28^ Proposed mechanisms of no-reflow—distal microembolization, endothelial swelling, capillary plugging by leukocytes and platelets, microvascular spasm, and oxidative injury—are well established in the literature.^29^ Although not directly examined, these processes are plausibly potentiated by the pro-atherogenic and pro-inflammatory milieu indexed by the PAI.^26^ Conceptually, the combination of impaired microvascular perfusion and reperfusion injury heightens dispersion of repolarization and facilitates triggered activity and reentry, lowering the threshold for sustained ventricular tachyarrhythmias during the index admission.

Measurement and interpretation considerations are relevant in acute care. Plasma atherogenic index is quickly derivable from routine lipid panels but depends on mmol/L units (with standard conversions from mg/dL) and may be influenced by acute-phase physiology, fasting status, and timing of sampling in ACS.^18^ These factors do not negate its utility but underscore the value of consistent pre-analytic practices and cautious interpretation. Plasma atherogenic index should be viewed as a complement to—not a substitute for—established lipoprotein measures such as LDL-C; where available, apoB or non-HDL-C quantify overall atherogenic particle burden and may be particularly informative in discordant phenotypes. Nevertheless, by jointly encoding TG-rich and HDL-related pathways, It can summarize aspects of the metabolic environment that single fractions may miss, especially when the endpoint of interest is electrical instability rather than purely atherosclerotic burden.

Positioning PAI relative to inflammatory and injury markers is also informative. The independent association for neutrophil burden accords with work linking neutrophil-dominant responses to arrhythmias and worse in-hospital events, plausibly via cytokine-mediated effects on ion channels, gap-junction remodeling, and reperfusion injury.^21^ Cardiac troponin, as an index of acute necrosis and a proxy for infarct size, is consistently related to downstream LVEF and electrical instability, aligning with its independent association here.^30^ Likewise, LVEF remains robust clinical anchors for risk stratification in malignant ventricular arrhythmias after MI, and multivessel disease plausibly augments substrate heterogeneity and reentry circuits through wider ischemic scar and conduction delays.^31^ Taken together, these patterns support integrating a PAI with readily available indicators of hemodynamic severity, injury, inflammation, global systolic function, and anatomic burden to guide the intensity of rhythm monitoring and targeted mitigation of reversible precipitants (ischemia relief, meticulous electrolyte optimization). Meta-analytic syntheses in coronary populations support the broader prognostic utility of PAI, strengthening the rationale to test whether adding PAI to arrhythmia-focused tools provides incremental discrimination and reclassification beyond standard markers.^32^

In addition to its association with malignant arrhythmias, the study demonstrated that higher PAI values were significantly related to increased in-hospital mortality, with deaths clustering in the upper tertile, Kaplan–Meier analysis confirming worse survival in patients with elevated PAI, and both regression and ROC analyses supporting its independent prognostic value. These findings align with previous reports showing that PAI is a strong predictor of adverse prognosis in acute coronary syndromes, including higher rates of mortality and major adverse cardiovascular events after PCI or medical management of ACS patients. Wu et al recently reported that adjusted PAI independently predicted major adverse cardiovascular events and mortality following PCI in STEMI patients^12^, while meta-analyses further support the prognostic role of PAI across different coronary populations.^18^ Collectively, these data suggest that PAI, as an easily obtainable lipid-based marker, may provide incremental prognostic value not only for arrhythmic complications but also for short-term survival in ACS.

Analytically, prioritizing PAI reduces redundancy and reflects an integrated lipid signal that captures elements of metabolic and atherogenic burden distinct from LDL-C, which retained an independent association when co-modeled. Although mechanistic pathways remain speculative without direct measures such as apoB, LDL particle metrics, or sdLDL, the findings suggest that PAI may complement traditional structural and hemodynamic risk markers in identifying patients vulnerable to malignant arrhythmias. Clinically, PAI offers practical advantages—it is inexpensive, universally available, and derived from routine triglyceride and HDL-C measurements obtained at admission—making its incorporation into early NSTEMI risk stratification feasible, particularly in resource-limited settings. Its potential to enhance monitoring intensity, guide therapeutic optimization, and provide incremental prognostic value alongside established scores merits further prospective evaluation.

This study has several limitations. The retrospective single-center design restricts causal inference and may limit generalizability to broader populations. Variability in rhythm-monitoring intensity and antiarrhythmic medication use could have influenced event detection. Lipid measurements may have been affected by acute-phase responses or non-fasting states, which could alter PAI values. Additionally, direct lipoprotein metrics such as apoB, LDL particle number/size, or sdLDL, were not included rendering mechanistic interpretations speculative. Prospective multicenter studies with standardized monitoring and formal evaluation of model performance—particularly discrimination, calibration, and reclassification metrics—are needed to refine the clinical utility of PAI in arrhythmia-oriented care pathways.

In this retrospective NSTEMI cohort, higher PAI values were independently associated with malignant ventricular arrhythmias during hospitalization, even after adjustment for conventional clinical, biochemical, and angiographic factors. Elevated PAI also demonstrated prognostic relevance for in-hospital mortality, with survival analyses and multivariable regression confirming its independent predictive value. Overall, these findings indicate that PAI—an inexpensive marker derived from routine lipid measurements—may serve as a practical tool for early risk stratification in NSTEMI, identifying patients at increased risk of electrical instability and short-term mortality. Prospective multicenter studies are needed to validate these observations and determine whether integrating PAI into clinical risk models can improve outcomes in acute coronary syndromes.

## Figures and Tables

**Table 1.** Comparison of Baseline Clinical, Laboratory, and Procedural Characteristics Between Patients with and without Malignant Arrhythmia

Characteristics	Patients Without Malign Arrhythmia, n = 455	Patients with Malign Arrhythmia, n = 47	*P*
Age (years)	59.76 ± 12.70	58.72 ± 13.13	.592
Male, n (%)	361 (79.3)	39 (83)	.555
Active smoking, n (%)	235 (51.6)	31 (66)	.061
Hypertension, n (%)	219 (48.1)	20 (42.6)	.466
Diabetes mellitus, n (%)	117 (25.7)	21 (44.7)	.006
CRF, n (%)	19 (4.2)	5 (10.6)	.048
PCI history, n (%)	89 (19.6)	14 (29.8)	.100
PAD, n (%)	23 (5.1)	4 (8.5)	.317
Family history, n(%)	135 (29.6)	10 (21.2)	.223
COPD, n(%)	21 (4.6)	4 (8.5)	.243
SBP, mmHg	137.63 ± 30.27	123.85 ± 37.18	.007
DBP, mmHg	77.72 ± 17.33	71.02 ± 19.55	.020
HR, beats/min	82.11 ± 19.99	93.37 ± 30.89	.001
KILLIP>1, n (%)	35 (7.6)	17 (36.2)	<.001
Multi-vessel disease, n (%)	143 (31.4)	23 (48.9)	.015
CAG result, n (%)	Medical, 28 (6.2) PCI, 379 (83.3) CABG, 26 (5.7) PCI+CABG, 22 (4.8)	Medical, 5 (10.6) PCI, 38 (80.9) CABG, 0 (0) PCI+CABG, 4 (8.5)	.162
Post-procedural no-reflow, n (%)	33 (7.3)	10 (21.3)	.001
Aspirin, n (%)	87 (19.1)	7 (14.9)	.479
ACEI/ARB, n (%)	151 (33.2)	15 (31.9)	.860
Beta-blocker, n (%)	98 (21.5)	11 (23.4)	.768
Statin, n (%)	129 (28.4)	14 (29.8)	.836
OAD, n (%)	100 (22)	21 (44.7)	.001
Hemoglobin (g/dL)	13.73 ± 1.98	13.95 ± 2.38	.472
White blood cells, (×10^9^/L)	11.40 (1.31-36.10)	12.50 (5.30-42.30)	.091
Platelets, (×10^9^/L)	225 (15-540)	235 (60-476)	.095
Creatinine (mg/dL)	0.84 (0.5-7.76)	1.04 (0.44-9.66)	.009
Estimated GFR (mL/min/1.73 m^2^)	97.78 (7.61-178.79)	76.58 (5.65-142.66)	.002
Sodium, mmol/L	136.99 ± 3.89	137.51 ± 5.72	.412
Potassium, mmol/L	4.22 ± 0.54	4.26 ± 0.80	.652
Magnesium, mmol/L	2.04 ± 0.30	2.15 ± 0.46	.154
Albumin g/dL	3.78 ± 0.39	3.75 ± 0.48	.681
C-reactive protein, mg/L	0.85 (0.20-33.10)	1.15 (0.20-31.50)	.996
Lymphocyte, 10^9^/L	1.80 (0.09-7.50)	1.92 (0.40-6.21)	.836
Neutrophil, 10^9^/L	8.50 (0.1-89.70)	9.94 (2.70-36.90)	.009
ALT (U/L)	26 (3-987)	29 (10-746)	.063
AST (U/L)	41 (3-1636)	36 (4-1704)	.857
TSH (mIU/L)	1.07 (0.01-91.81)	1.28 (0.06-16.63)	.100
Glucose (mg/dL)	128 (53-724)	176 (80-542)	<.001
HbA_1c_(%)	6.74 ± 2.20	7.76 ± 1.96	.008
Basal troponin T (ng/mL)	292 (50-5000)	1000 (48-14538)	<.001
LDL-C (mg/dl)	117 (40-276)	150 (21-239)	.001
HDL-C (mg/dl)	36 (5-82)	26 (16-55)	<.001
Triglyceride (mg/dl)	132 (35-873)	234 (73-809)	<.001
PAI	0.21 ± 0.29	0.48 ± 0.35	<.001
LVEF (%)	41.61 ± 8.75	35.42 ± 7.71	<.001
In-hospital mortality, n (%)	25 (5.5)	16 (34)	<.001

ACEI, angiotensin-converting enzyme inhibitor; ALT, alanine aminotransferase; ARB, angiotensin receptor blocker; AST, aspartate aminotransferase; CABG, coronary artery bypass grafting; CAG, coronary angiography; COPD, chronic obstructive pulmonary disease; CRF, chronic renal failure; CRP, C-reactive protein; DBP, diastolic blood pressure; GFR, glomerular filtration rate; HbA1c, hemoglobin A1c; HDL-C, high-density lipoprotein cholesterol; HR, heart rate; LDL-C, low-density lipoprotein cholesterol; LVEF, left ventricular ejection fraction; OAD, oral antidiabetic drug; PAD, peripheral arterial disease; PAI, plasma atherogenic index; PCI, percutaneous coronary intervention; SBP, systolic blood pressure; TSH, thyroid-stimulating hormone; WBC, white blood cell.

**Table 2.** Comparison of Clinical and Laboratory Characteristics Across Tertiles of Plasma Atherogenic Index (PAI: 27-75)

Characteristics	<0.027, n = 125	0.027-0.416, n = 252	>0.416, n = 125	*P*
Age (years)	60.35 ± 13.80	59.37 ± 12.29	59.59 ± 12.55	.779
Male, n (%)	94 (75.2)	205 (81.3)	101 (80.8)	.353
Active smoking, n (%)	61 (48.8)	129 (51.2)	76 (60.8)	.118
Hypertension, n (%)	64 (51.2)	121 (48)	54 (43.2)	.441
Diabetes mellitus, n (%)	27 (21.6)	71 (28.2)	40 (32)	.173
CRF, n (%)	4 (3.2)	16 (6.3)	4 (3.2)	.255
PCI history, n (%)	27 (21.6)	50 (19.9)	26 (20.8)	.928
PAD, n (%)	6 (4.8)	11 (4.4)	10 (8)	.320
Family history, n(%)	34 (27.2)	72 (28.7)	39 (31.2)	.778
COPD, n(%)	7 (5.6)	8 (3.2)	10 (8)	.120
SBP, mmHg	138.66 ± 29.12	137.58 ± 31.52	131.54 ± 32.25	.161
DBP, mmHg	78.20 ± 17.19 ^a*^	78.57 ± 17.38 ^a^	72.99 ± 18.09 ^b^	.017
HR, beats/min	81.00 ± 18.77	83.76 ± 22.03	84.15 ± 22.83	.425
KILLIP>1, n (%)	3 (2.4) ^a^	32 (12.9) ^b^	17 (13.6) ^b^	.003
Multi-vessel disease, n (%)	52 (41.6)	79 (31.3)	35 (28)	.052
CAG result, n (%)	Medical, 9 (7.2) PCI, 102 (81.6) CABG, 3.2 (5.7) PCI+CABG, 10 (8)	Medical, 16 (6.3) PCI, 213 (84.5) CABG, 6 (0) PCI+CABG, 8 (3.2)	Medical, 8 (6.4) PCI, 102 (81.6) CABG, 5.6 (0) PCI+CABG, 8 (6.4)	.453
Post-procedural no-reflow, n (%)	6 (4.8) ^a^	17 (6.7) ^a^	20 (16) ^b^	.002
Aspirin, n (%)	29 (23.2)	47 (18.7)	18 (14.4)	.204
ACEI/ARB, n (%)	48 (38.4)	83 (32.9)	36 (28)	.217
Beta-blocker, n (%)	23 (18.4)	56 (22.2)	30 (24)	.541
Statin, n (%)	34 (27.2)	77 (30.6)	32 (25.6)	.565
OAD, n (%)	28 (22.4)	58 (23)	35 (28)	.497
Hemoglobin (g/dL)	13.79 ± 1.93	13.75 ± 1.90	13.71 ± 2.32	.952
White blood cells, (×10^9^/L)	11.50 (1.31-26.10)	11.55 (1.80-36.10)	11.10 (5.51-42.30)	.311
Platelets, (×10^9^/L)	221 (15-384)	234 (104-540)	225 (80-476)	.143
Creatinine (mg/dL)	0.80 (0.60-4.86)	0.85 (0.55-9.66)	0.89 (0.44-6.55)	.101
Estimated GFR (mL/min/1.73 m^2^)	97.14 (9.80-148.71)	97.24 (5.65-178.79)	92.12 (9.89-142.83)	.553
Sodium, mmol/L	136.67 ± 4.36	137.04 ± 3.72	137.41 ± 4.51	.357
Potassium, mmol/L	4.21 ± 0.48	4.19 ± 0.51	4.31 ± 0.72	.141
Magnesium, mmol/L	2.00 ± 0.27	2.07 ± 0.31	2.07 ± 0.38	.175
Albumin g/dL	3.73 ± 0.43	3.76 ± 0.39	3.84 ± 0.39	.089
C-reactive protein, mg/L	1.60 (0.20-18.30)	1.20 (0.20-22.20)	0.80 (0.20-33.10)	.104
Lymphocyte, 10^9^/L	1.70 (0.38-7.50)	1.78 (0.09-6.83)	2.0 (0.18-5.80)	.836
Neutrophil, 10^9^/L	9.02 (0.54-23.30)	8.81 (0.1-32.90)	8.10 (3.20-89.70)	.168
ALT (U/L)	27 (3-987)	25 (8-228)	28 (8-746)	.456
AST (U/L)	47 (11-1636)	41 (3-739)	36 (4-1704)	.116
TSH (mIU/L)	1.06 (0.07-52.84)	1.15 (0.01-91.81)	1.10 (0.03-19.55)	.430
Glucose (mg/dL)	125 (67-514)	133 (53-724)	139 (74-542)	.119
HbA_1c_(%)	6.61 ± 1.74	6.82 ± 2.90	7.12 ± 1.77	.315
Basal troponin T (ng/mL)	347 (32-5000)	354 (5-14538)	332 (33-5000)	.864
LDL-C (mg/dl)	126 (40-229)	114 (42-276)	119 (21-239)	.206
HDL-C (mg/dl)	44 (25-82)^a^	36 (23-55)^b^	26 (5-49)^c^	<.001
Triglyceride (mg/dl)	79 (35-144)^a^	135 (68-268)^b^	249 (127-873)^c^	<.001
LVEF (%)	40.92 ± 8.58	41.20 ± 9.04	40.80 ± 8.74	.907
Patients with malign arrhythmia, n (%)	9 (7.2)^a^	8 (3.2)^a^	30 (24.4)^b^	<.001
In-hospital mortality, n (%)	3 (2.4)^a^	14 (5.6)^a^	24 (19.2)^b^	<.001

*Different superscript letters denote significant differences among groups.

ACEI, angiotensin-converting enzyme inhibitor; ALT, alanine aminotransferase; ARB, angiotensin receptor blocker; AST, aspartate aminotransferase; CABG, coronary artery bypass grafting; CAG, coronary angiography; COPD, chronic obstructive pulmonary disease; CRF, chronic renal failure; CRP, C-reactive protein; DBP, diastolic blood pressure; GFR, glomerular filtration rate; HbA1c, hemoglobin A1c; HDL-C, high-density lipoprotein cholesterol; HR, heart rate; LDL-C, low-density lipoprotein cholesterol; LVEF, left ventricular ejection fraction; OAD, oral antidiabetic drug; PAD, peripheral arterial disease; PAI, plasma atherogenic index; PCI, percutaneous coronary intervention; SBP, systolic blood pressure; TSH, thyroid-stimulating hormone; WBC, white blood cell.

**Table 3.** Univariable and Multivariable Logistic Regression Analysis for Predictors of Malignant Arrhythmia

	Univariable Regression		Multivariable Regression	
Variables	OR (%95 CI)	*P*	OR (%95 CI)	*P*
DM	2.333 (1.265-4.304)	.007	2.462 (0.976-6.213)	.056
SBP	0.985 (0.975-0.996)	.007	0.994 (0.981-1.008)	.401
KILLIP>1	6.735 (3.386-13.398)	<.001	1.634 (0.481-5.556)	.432
Multi-vessel disease	2.091 (1.142-3.830)	.017	2.726 (1.117-6.655)	.028
Post-procedural no-reflow	3.456 (1.579-7.564)	.002	2.033 (0.564-7.321)	.278
Estimated GFR	0.983 (0.974-0.993)	.001	0.992 (0.977-1.006)	.266
Neutrophil	1.066 (1.017-1.116)	.007	1.065 (1.015-1.118)	.010
Basal troponin T	1.004 (1.001-1.007)	<.003	1.000 (1.000-1.001)	.010
LDL-C	1.013 (1.006-1.021)	.001	1.023 (1.012-1.033)	<.001
PAI	1.252 (1.118-1.386)	<.001	1.228 (1.129-1.336)	<.001
LVEF	0.913 (0.877-0.951)	<.001	0.878 (0.824-0.934)	<.001

DM, diabetes mellitus; GFR, glomerular filtration rate; LDL-C, low-density lipoprotein cholesterol; LVEF, left ventricular ejection fraction; PAI, plasma atherogenic index; SBP, systolic blood pressure.

**Table 4.** Univariable and Multivariable Cox Regression Analysis for Predictors of Mortality

Variables	Univariable regression		Multivariable regression	
	HR (%95 CI)	*P*	HR (%95 CI)	*P*
DM	1.585 (0.813-3.092)	.176		
SBP	0.985 (0.974-0.996)	.007	0.994 (0.982-1.005)	.276
KILLIP>1	6.549 (3.191-13.439)	<.001	2.152 (0.732-6.326)	.164
Multi-vessel disease	1.327 (0.688-2.560)	.399		
Post-procedural no-reflow	4.916 (2.256-10.709)	<.001	2.748 (1.075-7.025)	.035
Estimated GFR	0.989 (0.979-1.000)	.044	1.002 (0.988-1.016)	.779
Neutrophil	1.039 (1.000-1.081)	.051		
Basal troponin T	1.002 (1.001-1.003)	.001	1.000 (1.000-1.001)	.009
LDL-C	0.999 (0.990-1.008)	.890		
PAI	1.315 (1.187-1.457)	<.001	1.342 (1.211-1.487)	.006
LVEF	0.967 (0.931-1.004)	.077		
Malignant arrhythmia	1.287 (1.162-1.426)	<.001	2.700 (1.014-7.193)	.047

DM, diabetes mellitus; GFR, glomerular filtration rate; LDL-C, low-density lipoprotein cholesterol; LVEF, left ventricular ejection fraction; PAI, plasma atherogenic index; SBP, systolic blood pressure.

**Table 5.** ROC Analysis for Predicting Malignant Arrhythmia and Mortality

ROC Analysis for Predicting Malignant Arrhythmia				
Variables	AUC	Asymptotic Sig.^b^	Asymptotic 95% CI	
			Lower Bound	Upper Bound
PAI	0.721	<0.001	0.626	0.815
Neutrophil	0.611	0.021	0.505	0.716
Basal troponin T	0.669	<0.001	0.597	0.740
LDL-C	0.658	0.001	0.554	0.762
LVEF	0.695	<0.001	0.610	0.780
ROC Analysis for Predicting Mortality				
PAI	0.709	<0.001	0.631	0.787
Basal troponin T	0.644	0.002	0.555	0.733

AUC, area under the curve; LDL-C, low-density lipoprotein cholesterol; LVEF, left ventricular ejection fraction; PAI, plasma atherogenic index.

## Data Availability

The data that support the findings of this study are available on request from the corresponding author.
